# Genetic background modulates the sensitivity of *Caenorhabditis elegans* to the toxic effects of vehicular air pollution

**DOI:** 10.17912/micropub.biology.000613

**Published:** 2022-07-21

**Authors:** Bailey A. Garcia Manriquez, Elise A. Kikis

**Affiliations:** 1 Biology Department, The University of the South, Sewanee, Tennessee, USA

## Abstract

Vehicular air pollution is an environmental toxicant that can have several health consequences, such as decreased respiratory and cardiovascular function and an increased incidence of age-related dementia and neurodegenerative diseases such as Alzheimer’s disease. *C. elegans* has been previously shown to be a valuable animal model to study the effects of air pollution due to its tendency to respond to acute exposure to nano-sized particulate matter (nPM) produced by vehicular emissions. Specifically, nPM causes delayed development resulting in smaller animal size and induction of the SKN-1-mediated oxidative stress response. Here we show that various wild isolates demonstrate differential susceptibility to nPM, as measured by body size. Specifically, the Hawaiian isolate, CB4856, displayed the highest sensitivity, equivalent to its sensitivity to the potent oxidant paraquat. The findings described herein suggest that *C. elegans* may be a useful genetic tool for identifying nPM-susceptibility loci.

**Figure 1. Genetic variation influences the susceptibility of C. elegans to the toxic effects of traffic-derived particulate air pollution:**
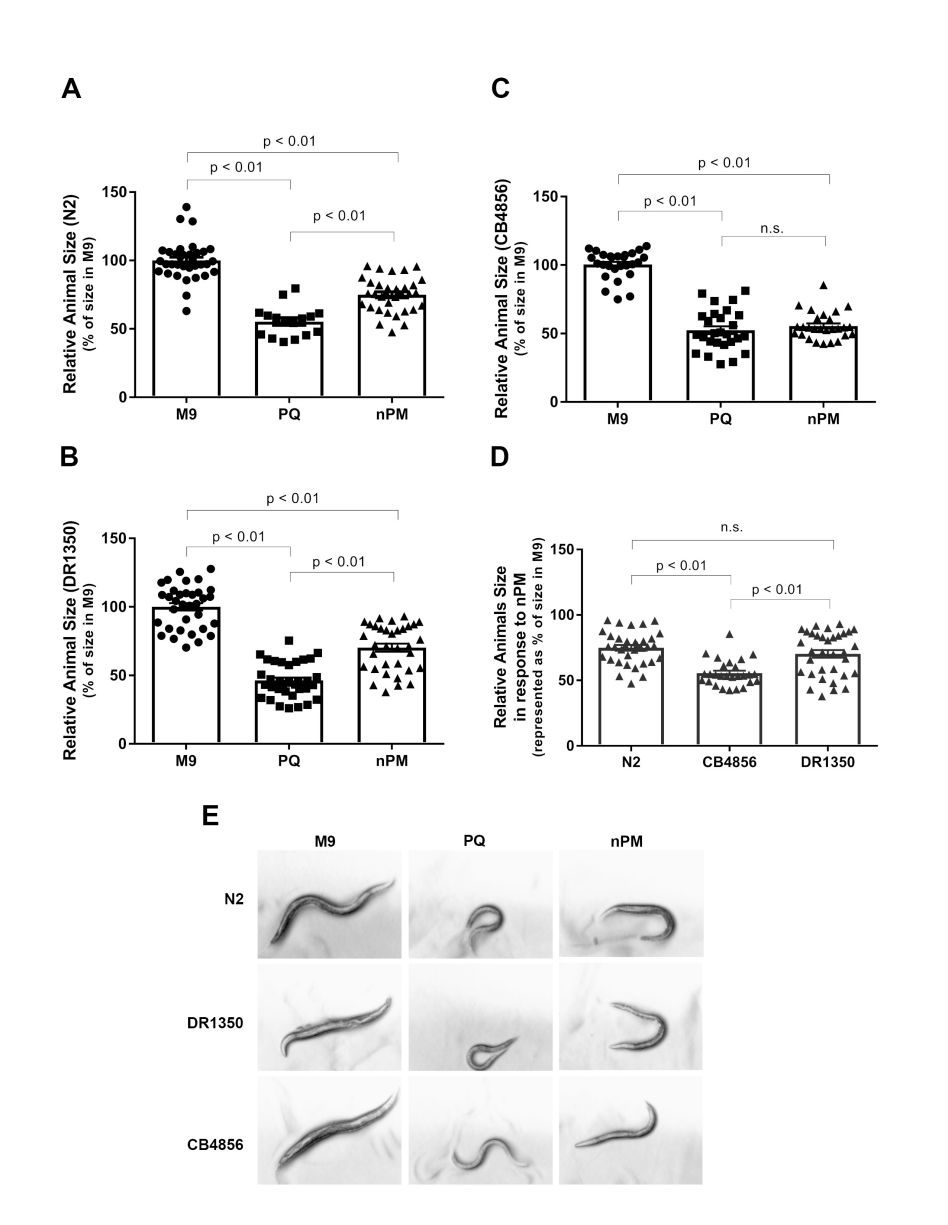
First larval stage (L1) N2 (A), DR1350 (B), or CB4856 (C) animals were either mock exposed (M9), exposed to paraquat (PQ), or exposed to traffic-derived nanoparticulate matter (nPM) for 1 h. The resultant body size after 3 d of recovery is indicated as a percent of the average size of animals exposed only to M9, which was set equal to 100%. D) To determine which genotype is most susceptible to nPM, the relative animal size of each genotype upon exposure to nPM was plotted side-by-side. For every graph, symbols represent the relative size of a single animal with n=16-34 individuals. Bars represent the mean relative animal size. Error bars represent the standard error of the mean. To identify statistically significant differences between samples, post hoc analyses were performed using the Bonferroni multiple comparisons test. P-values are indicated on graphs except in cases where the p-value was greater than 0.05 (not significant, n.s.). E) Representative micrographs of live animals 3 d after acute exposure, which were all taken at the same magnification.

## Description

Air pollution is an established risk factor for Alzheimer’s disease and age-related dementia (Cacciottolo et al., 2017; Shi et al., 2021) and its mechanism of action is under intense investigation. *C. elegans* is beginning to prove itself to be a valuable model organism to study the biological effects of traffic-derived particulate air pollution (Haghani et al., 2019). Specifically, *C. elegans* exposed in liquid to particulate air pollution, collected from a freeway in California, were shown to experience growth delay and oxidative stress-responsive gene expression (Haghani et al., 2019).

This prior study was performed exclusively in the N2 genetic background. However, gene—environment interactions, including interactions between air pollution and APOE gene variants, have been shown to contribute to cognitive decline in people (Schikowski et al., 2015). Therefore, we asked whether we could model the effects of genetic variation on susceptibility to air pollution in *C. elegans.* To address this question, we exposed three different wild isolates of *C. elegans* (N2, DR1350, and CB4856) to nano-sized particulate matter from traffic-derived air pollution (nPM). Additionally, because previous studies suggest that nPM induces oxidative stress (Cheng et al., 2016; Haghani et al., 2019; Cacciottolo et al., 2020), we exposed our animals to the powerful oxidant paraquat (PQ) as a positive control.

We found that 1 h of nPM exposure during development caused a statistically significant 25% reduction in the size of N2 animals, which is consistent with previous findings (Haghani et al., 2019). We also found that PQ had a similar but stronger effect, resulting in a 45% reduction in animal size **(Fig. 1A, E)** . Furthermore, both PQ and nPM caused statistically significant reductions (55% and 30%, respectively) in the size of DR1350 animals **(Fig. 1 B, E).** CB4856 animals experienced a 48% reduction in body size in response to PQ and a 45% reduction in response to nPM **(Fig. 1C, E)** . The difference between the response by CB4856 to nPM was not statistically different from the response to PQ **(Fig. 1C)** . Because all animals experienced a similar response to PQ, the enhanced sensitivity of CB4856 to nPM was likely not due to an increased susceptibility to oxidative stress. Importantly, with a 45% reduction in body size, CB4856 animals responded more strongly to nPM than did the other two wild type strains **(Fig. 1A-C)** . To better visualize the observed differential susceptibility to nPM, we re-plotted the N2, CB4856, and DR1350 animal size upon 1 h of exposure to nPM side-by-side and performed a Bonferroni multiple comparisons test. With p-values <0.01, we found that CB4856 was significantly smaller than either N2 or DR1350, which were comparable to one another **(Fig. 1D)** .

Taken together, the data presented herein suggest that CB4856 is particularly sensitive to nPM, but not hypersensitive to oxidative stress in general. This differential susceptibility to nPM is important because it recapitulates the sort of gene—environment interactions seen in people with respect to susceptibility to air pollution. Furthermore, it opens the door to future genetic studies using *C. elegans* to identify nPM-susceptibility loci.

## Methods

*C. elegans growth and maintenance*

All animals were maintained at 20 °C on nematode growth media (NGM) plates seeded with OP50 as a food source according to standard procedures (Brenner 1974).

*Exposures*

Exposures were performed in at least biological triplicate in 96 well plates with 10-15 first larval stage (L1) animals per well in a total volume of 200 µL. Paraquat (PQ) was diluted to a final concentration of 5mM in M9 and nanoparticulate matter from traffic-derived air pollution (nPM) was diluted to a final concentration of 75 µg/mL in M9. Mock treatments were performed with M9 alone. Exposures were for 1 h at 20 °C with gentle rocking on a nutator. Animals were then transferred to NGM plates for 3 d and grown at 20 °C prior to imaging.

*Imaging*

Live animals were imaged on NGM plates after cooling on ice to slow movement. Images of individual animals were obtained with a Leica DFC420 camera fitted to a Leica MZ16 stereomicroscope. Injured, dead, or bagged animals were excluded from further analysis.

*Quantification of animal size and statistics*

Animal size was determined from micrographs using the ImageJ software (version 1.8.0) and was normalized to mock exposed (M9) samples, whose mean was set equal to 100%. In the case of N2 animals exposed to PQ, the sample size was only n=16 due to many animals being sick or dead at the time of imaging. For all other genotypes and exposure conditions n>30. Statistical analyses and graphing were performed with the GraphPad Prism software (version 7). Post hoc analyses were performed using the Bonferroni multiple comparisons test and p ⩽ 0.05 was set as the statistical threshold for significance.

## Reagents

Nano-sized particulate matter (nPM) was obtained from traffic-derived air pollution collected near a highway in Los Angeles, CA as previously described (Haghani et al., 2019). As nPM batches may vary in their potency, the nPM used in this study was previously shown to be biologically active in *C. elegans* (Haghani et al., 2019).

The wild *C. elegans* isolates, N2 (Bristol), CB4856 (Hawaii), and DR1350 (California) were obtained from the *Caenorhabditis elegans* Genetics Center (Minneapolis, MN).
